# *In silico* analysis of highly conserved cytotoxic T-cell epitopes in the structural proteins of African swine fever virus

**DOI:** 10.14202/vetworld.2021.2625-2633

**Published:** 2021-10-09

**Authors:** Leana Rich De Mesa Herrera, Elizabeth Paulino Bisa

**Affiliations:** Department of Physical Sciences, College of Science, Polytechnic University of the Philippines, Manila, Philippines.

**Keywords:** African swine fever virus, cytotoxic T-cell epitopes, immunoinformatics, *in silico*

## Abstract

**Background and Aim:**

African swine fever (ASF) is a viral disease of pigs caused by ASF virus (ASFV). High mortality and the lack of available treatments have severely impacted the swine industry resulting in huge global economic losses. In response to the dire necessity for vaccines, this study aims to identify highly conserved cytotoxic T-cell epitopes in ASFV structural proteins pp220, pp62, p72, p30, and CD2v through immunoinformatics approach.

**Materials and Methods:**

The amino acid sequences of the structural proteins were retrieved from the National Center for Biotechnology Information protein database. The sequences were evaluated in CD-HIT Suite wherein resulting representative sequences were aligned in Clustal Omega. Highly conserved sequences were identified in the Protein Variability Server which were used as reference sequences for the cytotoxic T-cell epitope mapping. Epitopes were predicted using the tools in Immune Epitope Database. Peptides which bind to the swine major histocompatibility complex with IC_50_ binding scores >500 nM were filtered out. Epitopes which are classified to be potentially toxic and cross-reactive with the swine proteome sequences were all excluded from the study. The epitopes were docked with the swine leukocyte antigen-1*0401 (SLA-1*0401) wherein the binding affinity, the binding energy, and the root-mean-square deviation (RMSD) per residue of epitope-SLA complexes formed were determined and compared with the influenza epitope as positive control.

**Results:**

A total of 112 highly conserved fragments with Shannon variability index ≤0.1 were identified. These include 66, 12, 26, 6, and 2 highly conserved fragments from ASFV proteins pp220, pp62, p72, p30, and CD2v, respectively. From these reference sequences, 35 nonameric peptides were selected for the list of candidate cytotoxic T-cell epitopes. These include 26 epitopes for pp220, 7 for pp62, 6 for p72, and one each for p30 and CD2v. Bioinformatics analysis classified the peptides as non-toxic. Further evaluations of epitopes showed that these are less likely to cross-react with the domestic swine proteome sequences. This study identified candidate epitopes from pp220 (IADAINQEF, FLNKSTQAY, QIYKTLLEY, and SLYPTQFDY), and pp62 (GTDLYQSAM, FINSTDFLY, and STDFLYTAI) which can bind to at least two widely distributed SLAs in pig populations. The immunogenicity of candidate peptides RSNPGSFYW, DFDPLVTFY, AIPSVSIPF, and VVFHAGSLY was validated by the acceptable binding affinities, binding energies, and RMSD of the peptide-SLA complexes formed. Results were also comparable with the crystal structure of an SLA-epitope complex in the database.

**Conclusion:**

This is the first study to identify highly conserved cytotoxic T-cell epitopes in the structural proteins of ASFV. Overall, the results of *in silico* evaluations showed that the identified highly conserved cytotoxic T-cell epitopes may be used as part of future vaccine formulations against ASFV infection in domesticated pigs. Nonetheless, these findings require *in vitro* and *in vivo* validation before application.

## Introduction

African swine fever (ASF) is a viral disease of pigs with mortality approaching 100% [1]. It was first identified in Kenya in the 1920s; then, it had spread to Europe in the middle of the past century. In 1990s, ASF was eradicated from the most affected regions (except Sardinia and sub-Saharan countries) due to the implementation of biosafety regulations. But in 2007, ASF rapidly spread out again from Africa. At present, ASF has been identified in Africa, Europe, America, and Asia [2-4]. ASF is caused by the ASF virus (ASFV) which can spread between soft ticks (*Ornithodoros erraticus*) and wild pigs through feeding, or less frequently through direct transmission between wild pigs. In domesticated pigs, ASFV can be readily transmitted resulting to lethal hemorrhagic fever. The widespread of ASF has been a great challenge for swine breeding. Although it does not pose a risk to human health, ASF has resulted to huge economic losses around the world [5]. ASFV, the only species of the genus *Asfivirus* within the family *Asfarviridae*, is an icosahedral enveloped virus with double-stranded DNA as its genetic material. The viral particle consists of an inner core coated by a thick protein core shell enclosing its genetic material. The subsequent layers are the lipid envelope surrounding the protein core shell, and the viral capsid which forms the outermost cover of an intracellular virion. Virions budding out of the host cell carry a portion of the host plasma membrane forming an extra layer of envelope [6]. The length of its genome differs from one isolate to another (170-194 kbp) with 151-167 open reading frames [7]. ASFV encodes around 50 structural proteins with significant roles in genome replication and viral packaging. Vital structural proteins include pp220, pp62, p72, p30, and CD2v. These major components of viral particles are important in viral attachment, entry, replication, and processing [8]. Both pp220 and pp62 are polyprotein precursors proteolytically cleaved to mature virion proteins. Structural polyprotein pp220 is encoded by gene *CP2475L* which product is cleaved and post-translationally processed to p150, p37, p14, and p34. Structural protein pp62, a relatively shorter polypeptide, is encoded by gene *CP530R*. Similar to pp220, this polyprotein is proteolytically cleaved into mature p35 and p15 virion proteins. The post-translational products of pp220 and pp62 play roles in viral particle assembly and form the major component of the viral core shell [9,10]. Findings suggest that the expression of the major capsid protein p72 is a requirement for the processing of pp220 and pp62 in ASFV [11]. Antigenic structural protein p72 is encoded by gene *B646L* (*VP72*) which serves as a major protein component in viral capsids and functions in the formation of ASFV capsids [12]. Another crucial structural protein in ASFV is p30. It is encoded by *CP204L* gene and is most abundantly expressed during the early phase of infection. It has crucial functions in viral entry and is known to be one of the most antigenic ASFV proteins [13,14]. Studies showed that recombinant p30 can be an efficient ASFV antibody detector both in oral fluid and serum samples [15]. ASFV gene *EP402R* encodes the structural glycoprotein CD2v which consists of transmembrane region, extracellular domain (N-terminal), and cytosolic domain (C-terminal). CD2v is associated to immune response modulation and lymphocyte function impairment which enhance the virulence of ASFV in domestic swine [16]. In addition, it has been shown to be directly involved in viral hemadsorption resulting in increased spread of the virus [17].

Due to the significant roles of pp220, pp62, p72, p30, and CD2v in ASFV replication and assembly, disporting key properties including immunogenicity, abundant expression, and virulence mechanism, these structural proteins can be suitable immunotherapeutic targets in designing vaccines and treatments against ASFV. At present, there are no anti-viral agents and vaccines available against ASFV [5]. The significant economic diminution brought about by the ASFV infection in swine populations around the world warrants accelerated vaccine development.

In the pursuit of this compelling demand, the researchers utilized immunioinformatics approach to provide potential immunotherapeutic agents against ASFV. Immunoinformatics tools and databases can aid in hastening and reducing the cost required to identify immunogenic proteins and peptides for vaccine designs [18,19]. Identification of epitopes through a computational approach is an advantageous and prototypical step in the process of vaccine development before any further *in vitro* and *in vivo* evaluations are conducted.

Thus, this study aims to identify cytotoxic T-cell epitopes from the conserved amino acid sequences of pp220, pp62, p72, p30, and CD2v in ASFV. The use of highly conserved sequences in vaccine development is one of the most important factors that must be considered to resolve immune epitope evasion in the rapidly mutating viral antigens.

## Materials and Methods

### Ethical approval

Ethical approval is not necessary for this type of study.

### Study period and location

The study was conducted from April to June 2021. The study was conducted at the Polytechnic University of the Philippines.

### Retrieval and identification of highly conserved sequences

ASFV protein sequences of pp220, pp62, p72, p30, and CD2v were retrieved from the National Center for Biotechnology Information protein database on April 17, 2021. Search results were filtered using 2475-2476, 530-534, 645-646, 203-204, and 360-404 for the sequence length of pp220, pp62, p72, p30, and CD2v, respectively. The lists of representative sequences for each antigen were obtained using CD-HIT Suite (http://weizhongli-lab.org/cdhit_suite/cgi-bin/index.cgi?cmd=cd-hit) with 1.00 as sequence-identity cutoff. Unique representative sequences for each protein were aligned in Clustal Omega (https://www.ebi.ac.uk/Tools/msa/clustalo/) to generate multiple sequence alignment. To identify highly conserved sequences for each protein, Shannon variability threshold H ≤0.1 was used in the protein variability server (PVS) tool (http://imed.med.ucm.es/PVS/). The first sequence in the alignment was used as a reference and the varying residues were masked. Fragments with ≥9 residues were kept for cytotoxic T-cell epitope identification.

### Cytotoxic T-cell epitope mapping

Cytotoxic T-cell epitopes which can bind to widely distributed swine leukocyte antigens (SLA) SLA-1*0101, SLA-1*0401, and SLA-1*0801 in swine population [20] were mapped in the Immune Epitope Database. NetMHCcons was utilized to integrate three well-known methods: NetMHC, NetMHCpan, and PickPocket to produce more reliable results [21]. All binders with TAP and proteasome scores below zero were excluded from the study. Epitopes with major histocompatibility complex (MHC) IC_50_ <500 nmol/dm^3^ are classified as good binders [22]; thus, these were further evaluated for cross-reactivity and toxicity. Protein-protein BLAST (BLASTp) was utilized to identify and exclude epitopes with a significant match to swine protein sequences. Sequence match with e-values <1.0e-30 can be cross-reactive in some allergic individuals [23]. Although this result was found in humans, it was adopted in this work to provide a more precise cutoff reference in the identification of potentially cross-reactive peptides. Epitopes were queried against the toxin peptide database in ToxinPred tool (https://webs.iiitd.edu.in/raghava/toxinpred/multi_submit.php) to identify potentially toxic peptides that can cause damage to cells through the default SVM Method [24]. Peptides which overlap with cleavage and glycosylation sites in each protein sequence were excluded from the study. All remaining peptides were included as candidate cytotoxic T-cell epitopes.

### SLA-epitope docking and molecular dynamics

The crystal structure of SLA-1*0401 bound to influenza epitope (PDB ID: 3QQ3) was retrieved from the Research Collaboratory for Structural Bioinformatics Protein Data Bank (RCSB PDB). The PDB file was cleaned to obtain the isolated structure of SLA-1*0401 for subsequent docking procedures. For each structural antigen, epitope with the highest IC_50_ binding affinity for SLA-1*0401 was docked with the cleaned PDB structure of SLA-1*0401 in GalaxyPepDock server (http://galaxy.seoklab.org/cgi-bin/submit.cgi?type=PEPDOCK). The resulting top score structures of SLA-epitope complex were further refined in the GalaxyRefineComplex server (http://galaxy.seoklab.org/cgi-bin/submit.cgi?type=COMPLEX). Refined SLA-epitope structures were viewed using the iCn3D tool (https://www.ncbi.nlm.nih.gov/Structure/icn3d/full.html). The dissociation constant (K_d_) and the binding energy (∆G_bind_) of each SLA-epitope complex were calculated in PRODIGY (https://bianca.science.uu.nl/prodigy/) at 300 K. This webserver tool uses non-interface surface properties and intermolecular contacts for predictive models [25]. Estimated K_d_ and ∆G_bind_ values for SLA-1*0401-bound epitopes were compared with that of the influenza epitope bound to SLA-1*0401. To determine the stability of SLA-epitope complex formation, molecular dynamic simulation was performed in MDWeb server (http://mmb.irbbarcelona.org/MDWeb/) to obtain the root-mean-square deviation (RMSD) plot per residue. Molecular dynamics parameters include C-alpha Brownian dynamics in 100 ps time, 0.01 ps time change, 3.8 Ǻ distance between alpha carbon atoms, 10 step output frequency, and 167.36 kJ/mol Ǻ^2^ force constant. This process employs the GROMACS MD setup with solvation using force-field Amber-99sb [26].

## Results

### Highly conserved sequences in the ASFV structural proteins

Herein, the reference sequences identified for the ASFV structural proteins pp220, pp62, p72, p30, and CD2v have Shannon variability index ≤0.1. Sequences of pp220, pp62, p72, p30, and CD2v yielded 66, 12, 26, 6, and 2 highly conserved fragments, respectively. Table-1 shows the positions of highly conserved fragments identified for each antigen. Conserved sequences in pp220, pp62, and p72 are widely distributed as indicated by the presence of longer fragments adjacent to each other within the full stretch of their amino acid sequences. Antigens pp220, pp62, and p72 contain highly conserved fragments with lengths ranging from 9 to 102, 11 to 139, and 9 to 51, respectively. All these highly conserved fragments were used as reference sequences for cytotoxic T-cell mapping.

**Table 1:** Highly conserved sequences in ASFV structural proteins.

ASFV antigen	Start	End	Sequence
pp220	1	13	M G N R G S S T S S R P P
	21	68	Y A K L Q D H I Q R Q T R P F S G G G Y F N G G G D K N P V Q H I K D Y H I D S V S S K A K L R
	85	119	D T K Q P I E D I L K D I K K Q L P D P R A G S T F V K N A E K Q E T
	121	144	C K M I A D A I N Q E F I D L G Q D K L I D T T
	146	186	G A A S I C R Q I V L Y I N S L T H G L R A E Y L D V H G S I E N T L E N I K L L
	188	232	D A I K Q L H
			E R M V T E V T K A A P N E E V I N A V T M I E A V Y R R L L N E Q N L Q I
	234	253	I L T N F I D N I L T P T Q K E L D K L
	264	291	L N D T N S V L G T K N F G K V L S Y T L C N L G I A A
	293	302	V A N K I N K A L Q
	304	312	V G L K V E Q Y L
	319	361	E F D K E L D L K R F S G L V S A E N I A E F E K A V N L L R Q T F N E R H K I L E N
	380	412	E A Q R L D R K H I L M E F L N K S T Q A Y N D F L E N V K K I G
	414	426	K L V K E I A L T P N I T
	428	451	L R D A L S R I N D M G T I A L D L S L I G F Y
	453	464	N A A A R E E R E T F L
	481	491	D P N F K N L Y D S C
	493	511	R L L Q I I D F Y T D I V Q K K Y G G
	520	620	V G G A A L T V E E L G L S K A A R S Q V D L N Q A I N T F M Y Y Y Y V A Q I Y S N L T H N K Q E F Q S Y E E N Y A T I L G D A I A G R L M Q L D T E K N A R I N S P A V D L A R G H V G P N P G G A Q E
	628	729	S A I E L E Y D V K R R F Y R A L E G L D L Y L K N I T K T F V N N I D S I Q T V Q Q M L D G V R I I G R W F T E A T G D T L A Q V F E S F P T S A G N D S N V F T D N A P A G H Y Y E K V A A E I Q Q G R
	731	780	V G T L R P V R A S Q A K N I R D L I G R S L S N F Q A L K N I I N A F A R I G D M L G G E E L R Q
	782	796	V P M S P L Q I Y K T L L E Y
	798	811	Q H S A L S V G L K N L N Q
	830	844	Q R V Y L S T V R V N D A L S
	853	906	F F T F M L K S M A A K I F I V L G I Y D M F E R P E P V Y K L I P T R M I L G G A D E L E P E V I P E A A
	908	927	L Y F R L P R L A E F Y Q K L F S F R D
	929	947	N V Q I S M L P E L E G I F S G L I R
	949	990	I F M R P I E L I N I G D Y S E T E I R Q L I K E I N V I Y Q H F N L E Y G E Q E A
	992	1060	K K A L I H F V N E I N R R F G V I T R T E W E K F Q R I V Q E A R T M N D F G M M N Q T N Y S I L P D E D G Y T Q S S Q L L P S D R F I
	1065	1118	Q P T P K W R P A L Y N I D S V D V Q T G M L Q P N S Q W D L V Q K F R K Q L S E M F E D P S L Q Q E L G K
	1136	1183	H T D K I Q I V S K L I Q G S E S L A D T D V N K I F L F H E T V I T G L N L L S A I Y V L L N
	1192	1208	L D L D T I Q K S I I E W L R E T
	1229	1238	I S E I R N P G L V
	1289	1313	E Q E L A A R Y L V D N Q R I M Q L L L T N I F E
	1323	1332	Q V R F P E T S T A
	1334	1356	V H L D F T G L I S L I D S L M A D T K Y F L
	1358	1398	L L R P H I D K N I I Q Y Y E N R S N P G S F Y W L E E H L I D K L I K P P T D A
	1400	1421	G R P L P G G E L G L E G V N Q I I N K T Y
	1423	1433	L L T K P Y N V L Q L
	1442	1451	A A N I Q I N N N P
	1476	1484	N S G L R V E Q V
	1486	1504	L G D F R L S N L I R T N N A Q E E N
	1517	1536	Y A N V N D A A N N L R R Y R L Y G S D
	1541	1552	N N R S M M M V F N Q L
	1559	1574	R F Y D A P S G K I Y L N L I N
	1576	1587	F A N G N F S Q A V M E
	1601	1620	A F G H R G D P T E Q S V L L L S L G L
	1622	1681	L Q R L I K D T N R Q G L S Q H L I S T L T E I P I Y L K E N Y R A N L P L F N K M F N I L I S Q G E L L K Q F I Q Y T
	1770	1784	S A M E V L H E L T D H P I Y
	1786	1841	E T E E H F I Q N Y M S R Y N K E P L M P F S L S L Y Y L R D L R I E N N E V Y D P L L Y P N L E S G S P E F K
	1843	1876	L Y G T R K L L G N D P V Q L S D M P G V Q L I M K N Y N E T V V A
	1878	1889	E Q I T P T R F E H F Y
	1891	1899	H A I Q A L R F I
	1901	1909	N I R S F K T V M
	1911	1922	Y N E N T F G G V N L I
	1935	1965	G I G M N A V Y S L R K T L Q D V I S F V E S S Y Q E E Q I N
	1980	1997	L G S N R E R E R I F N L F D M N I
	1999	2039	P I N V N A L M R S I P L A N I Y N Y D Y S F E E I A C L M Y G I S A E K V R S L
	2051	2059	V L N I P N R P P
	2061	2075	N T R E F M L K L L I N P Y V
	2077	2086	V S I T Q Y G N E L
	2092	2138	A G Y M S R I F R G D N A L N M G R P K F L S D Q I F N K V L F G S L Y P T Q F D Y D E A G P
	2140	2149	L A A G I Q R G R E
	2380	2397	E E G P W S I V K Q V G V G I Q K P
	2402	2420	I G K D R F D T R L I R N L I F I T N
	2422	2443	Q R L L R L R L N L E L S Q F R N V L V S P
	2444	2476	H I I N P S I T E Y G F S I T G P S E T F S D K Q Y D S D I R I L
pp62	1	86	M P S N M K Q F C K I S V W L Q Q H D P D L L E I I N N L C M L G N L S A A K Y K H G V T F I Y P K Q A K I R D E I K K H A Y S N D P S Q A I K T L E S L I L P F Y I P T P
	88	105	E F T G E I G S Y T G V K L E V E K
	107	120	E A N K V I L K N G E A V L
	122	164	P A A D F K P F P D R R L A V W I M E S G S M P L E G P P Y K R K K E G G G N D P P V
	166	186	K H I S P Y T P R T R I A I E V E K A F D
	188	253	C M R Q N W C S V N N P Y L A K S V S L L S F L S L N H P T E F I K V L P L I D F D P L V T F Y L L L E P Y K T H G D D F L I P E T
	255	291	L F G P T G W N G T D L Y Q S A M L E F K K F F T Q I T R Q T F M D I A D
	293	312	A T K E V D V P I C Y S D P E T V H S Y
	314	324	N H V R T E I L H H N
	326	464	V N K V T T P N L V V Q A Y N E L E Q T N T I R H Y G P I F P E S T I N A L R F W K K L W Q D E Q R F V I H G L H R T L M D Q P T Y E T S E F A E I V R N L R F S R P G N N Y I N E L N I T S P A M Y G D K H T T G D I A P N D R F A M L V A F I N S T D F L Y T A I P E E K V G G N
	472	504	T S S L T D L V P T R L H S F L N H N L S K L K I L N R A Q Q T V
	506	530	N I L S N D C L N Q L K H Y V K H T G K N E I L K
p72	25	34	L L N S R I S N I K
	36	45	V N K S Y G K P D P
	58	69	V H F N A H F K P Y V P
	71	91	G F E Y N K V R P H T G T P T L G N K L T
	94	108	I P Q Y G D F F H D M V G H H
	110	124	L G A C H S S W Q D A P I Q G
	134	184	L Q T F P R N G Y D W D N Q T P L E G A V Y T L V D P F G R P I V P G T K N A Y R N L V Y Y C E Y P G
	195	210	V N G N S L D E Y S S D V T T L
	212	244	R K F C I P G D K M T G Y K H L V G Q E V S V E G T S G P L L C N
	251	268	P H Q S K P I L T D E N D T Q R T C
	270	281	H T N P K F L S Q H F P
	286	300	N I Q T A G K Q D I T P I T D
	302	310	T Y L D I R R N V
	318	331	Q T P K Y Y Q P P L A L W I
	340	375	N V N L A I P S V S I P F G E R F I T I K L A S Q K D L V N E F P G L F
	384	402	G R P S R R N I R F K P W F I P G V I
	410	426	N E L Y I N N L F V T P E I H N L
	428	454	V K R V R F S L I R V H K T Q V T H T N N N H H D E K
	456	487	M S A L K W P I E Y M F I G L K P T W N I S D Q N P H Q H R D W
	489	500	K F G H V V N A I M Q P
	507	525	S F Q D R D T A L P D A C S S I S D I
	529	555	T Y P I T L P I I K N I S V T A H G I N L I D K F P S
	557	565	F C S S Y I P F H
	588	611	P R E E Y Q P S G H I N V S R A R E F Y I S W D
	614	626	Y V G S I T T A D L V V S
	627	645	S A I N F L L L Q N G S A V L R Y S T
p30	22	33	S S Q V V F H A G S L Y
	39	55	E I I N S G R I V T T A I K T L L
	78	94	Q A Q E E W N M I L H V L F E E E
	121	136	E C T S S F E T L F E Q E P S S
	153	165	V Q H I E Q Y G K A P D F
	191	201	V R L M V I K L L K K
CD2v	296	304	K H V E E I E S P
	322	351	S I H E P S P R E P L L P K P Y S R Y Q Y N T P I Y Y M R P

ASFV=African swine fever virus

### Highly conserved cytotoxic T-cell epitopes in the structural proteins of ASFV

All resulting cytotoxic T-cell epitopes mapped using the reference sequences were filtered by excluding potentially toxic and cross-reactive peptides. Table-2 shows the list of candidate cytotoxic T-cell epitopes in pp220, pp62, p72, p30, and CD2v indicating their corresponding SLA binders, sequence location, proteasome score, TAP score, and MHC IC_50_. A total of 35 highly conserved epitopes from the five structural proteins of ASFV were identified. These include 26 peptides from pp220, 7 from pp62, 6 from p72, and one each from p30 and CD2v. Analysis revealed that seven of these epitopes (four in pp220 and three in pp62) can bind to at least two widely distributed SLAs. In this study, all candidate cytotoxic T-cell epitopes have MHC IC_50_ ≤500 nmol/dm^3^ with positive TAP and proteasome scores.

**Table 2:** Highly conserved cytotoxic T-cell epitopes in ASFV.

ASFV antigen	SLA	Start	End	Epitope	Proteasome score	TAP score	MHC IC_50_
pp220	SLA-1*0401	124	132	IADAINQEF	1.43	1.07	50.2
	SLA-1*0101	124	132	IADAINQEF	1.43	1.07	226
	SLA-1*0101	132	140	FIDLGQDKL	1.51	0.28	474.3
	SLA-1*0401	213	221	AVTMIEAVY	1.43	1.44	363.9
	SLA-1*0401	344	352	AVNLLRQTF	1.51	1.23	276.1
	SLA-1*0401	383	391	RLDRKHILM	1.05	0.15	89.1
	SLA-1*0401	393	401	FLNKSTQAY	1.51	1.12	69.9
	SLA-1*0801	393	401	FLNKSTQAY	1.51	1.12	226
	SLA-1*0401	442	450	ALDLSLIGF	1.53	1.1	30.9
	SLA-1*0401	501	509	YTDIVQKKY	1.5	1.13	53
	SLA-1*0401	545	553	AINTFMYYY	1.38	1.33	157.3
	SLA-1*0101	682	690	FTEATGDTL	1.66	0.28	238.6
	SLA-1*0401	788	796	QIYKTLLEY	1.45	1.43	73.4
	SLA-1*0801	788	796	QIYKTLLEY	1.45	1.43	309.4
	SLA-1*0401	1079	1087	SVDVQTGML	1.38	0.36	173.4
	SLA-1*0401	1362	1370	HIDKNIIQY	1.48	1.15	28.9
	SLA-1*0401	1374	1382	RSNPGSFYW	1.16	0.53	392.5
	SLA-1*0401	1791	1799	FIQNYMSRY	1.26	1.26	180.1
	SLA-1*0401	1832	1840	NLESGSPEF	1.41	1	257.4
	SLA-1*0101	1857	1865	LSDMPGVQL	1.49	0.34	317.8
	SLA-1*0401	2104	2112	ALNMGRPKF	1.15	1.19	238.6
	SLA-1*0401	2125	2133	SLYPTQFDY	1.02	1.37	112.5
	SLA-1*0801	2125	2133	SLYPTQFDY	1.02	1.37	222.4
	SLA-1*0401	2446	2454	IINPSITEY	1.33	1.28	67.3
pp62	SLA-1*0401	73	81	TLESLILPF	1.11	1.08	202.9
	SLA-1*0101	138	146	IMESGSMPL	1.01	0.39	454.2
	SLA-1*0401	227	235	DFDPLVTFY	1.36	1.13	324.8
	SLA-1*0401	263	271	GTDLYQSAM	0.78	0.03	145.1
	SLA-1*0101	263	271	GTDLYQSAM	0.78	0.03	256
	SLA-1*0801	445	453	FINSTDFLY	1.2	1.31	386.2
	SLA-1*0401	445	453	FINSTDFLY	1.2	1.31	73.4
	SLA-1*0101	448	456	STDFLYTAI	0.98	0.23	98.8
	SLA-1*0401	448	456	STDFLYTAI	0.98	0.23	180.1
	SLA-1*0101	475	483	LTDLVPTRL	1.43	0.36	346.6
p72	SLA-1*0401	153	161	AVYTLVDPF	0.83	1.29	348.4
	SLA-1*0101	157	165	LVDPFGRPI	0.83	0.22	346.6
	SLA-1*0401	199	207	SLDEYSSDV	0.79	0.15	373.8
	SLA-1*0401	233	241	SVEGTSGPL	0.95	0.46	375.9
	SLA-1*0401	344	352	AIPSVSIPF	0.97	1.23	384.1
	SLA-1*0401	599	607	NVSRAREFY	1.24	1.35	258.8
p30	SLA-1*0401	25	33	VVFHAGSLY	1.28	1.38	132.3
CD2v	SLA-1*0801	340	348	YQYNTPIYY	1.28	1.43	245.2

SLA=Swine leukocyte antigen, ASFV=African swine fever virus

### Validation of identified cytotoxic T-cell epitopes

**Figure-1:**
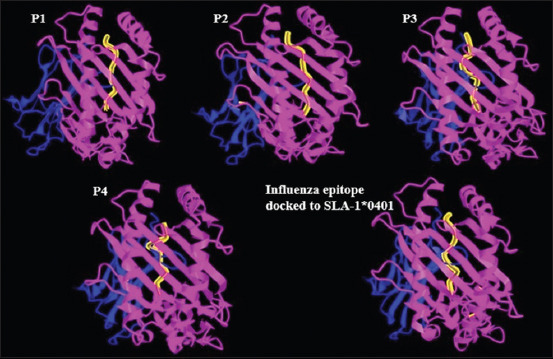
Cytotoxic T-cell epitopes docked to the binding groove of swine leukocyte antigen-1*0401.

The PDB structure of SLA-1*0401 was utilized for the validation procedures in this study because the crystal structures of the other two SLAs (0101 and 0801) are not available in the RCSB protein data bank. Thus, for every antigen, the epitope with the lowest binding affinity (with the highest IC_50_) to SLA-1*0401 was employed for the docking procedures. The candidate epitope YQYNTPIYY in CD2v binds to SLA-1*0801; thus, it was not included in the docking procedures. Peptides docked to SLA-1*0401 include pp220 RSNPGSFYW (P1) with MHC IC_50_ 392.5, pp62 DFDPLVTFY (P2) with MHC IC_50_ 324.8, p72 AIPSVSIPF (P3) with MHC IC_50_ 384.1, and p30 VVFHAGSLY (P4) with MHC IC_50_ 132.3. The influenza epitope NSDTVGWSW bound to SLA-1*0401 was used as a control to serve as a supporting reference for positive binding. Figure-1 shows the 3D structures of complexes formed for P1, P2, P3, and P4 and influenza epitopes (yellow), with the swine MHC I (magenta and blue) binding groove.

**Figure-2:**
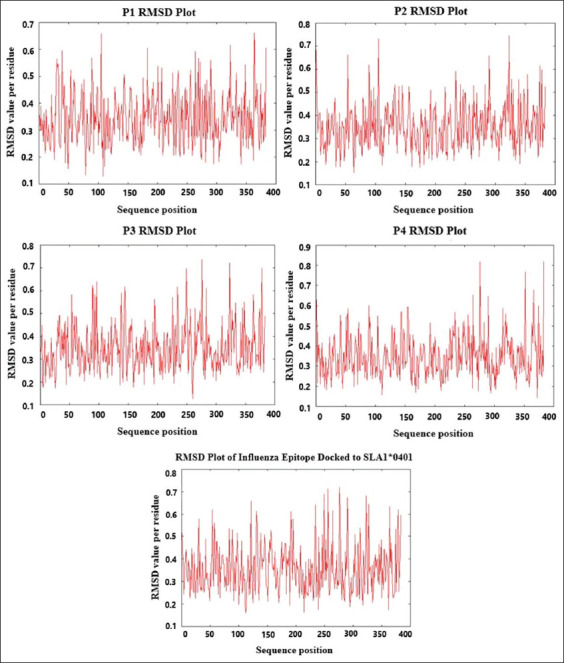
Root-mean-square deviation plots for the residues of swine leukocyte antigen-epitope complexes.

To evaluate if the binding of epitopes to SLA-1*0401 is favorable, the dissociation constant(K_d_) and the binding energy (DG_bind_) of each SLA-epitope complex formed were calculated. Table-3 shows that the binding of candidate epitopes RSNPGSFYW (P1), DFDPLVTFY (P2), AIPSVSIPF (P3), and VVFHAGSLY (P4) with SLA-1*0401 resulted in negative DG_bind_ values with very small K_d_. Notice that the DG_bind_ and K_d_ values of identified cytotoxic T-cell epitopes in this study are comparable to the influenza epitope-SLA complex. Figure-2 shows the plot of sequence position and the RMSD per residue for each complex formed by SLA-1*0401 with P1, P2, P3, and P4, and influenza epitopes. All RMSD values in each complex are lower than 0.9.

**Table 3:** Binding energy and binding affinity of SLA-epitope complexes.

Epitope docked to SLA-01*0401	∆G_bind_ (kJ/mol)	K_d_ (mol/dm^3^)	Antigen source
RSNPGSFYW	−46.86	1.30E-08	pp220
DFDPLVTFY	−46.86	1.30E-08	p62
AIPSVSIPF	−42.26	7.50E-08	p72
VVFHAGSLY	−45.61	2.10E-08	p30
NSDTVGWSW	−51.04	2.60E-09	Influenza epitope

SLA=Swine leukocyte antigen

## Discussion

The recent emergence of ASFV in new countries and continents has significantly impacted worldwide pork production due to its high mortality [27]. At present, there are no commercially available vaccines [28] to treat or prevent domestic swine from this pathogen. Herein, this work aims to identify peptides that can be incorporated as part of a potential vaccine, targeting immunogenic proteins with crucial roles in the virulence of ASFV. Due to higher mutation rates of viral genomic materials, one of the most important properties in vaccines design is the eluding of immune epitope escape by utilizing highly conserved sequences for the identification of epitopes. This strategy may enhance efficacy and longer-term immunity against viral antigens. Therefore, this study utilized highly conserved reference sequences for cytotoxic T-cell epitope mapping. The Shannon variability index of ≤0.1 for all the identified fragments indicates that the reference sequences are highly conserved [29,30]. Cytotoxic T-cell response is critical for effective resolution of viral infections. It has been suggested that the cytotoxic T lymphocytes have important roles in ASFV protective immunity [1,31]. This study identified cytotoxic T-cell epitopes binding to the widely distributed SLA in pig populations which include SLA-1*0101, SLA-1*0401, and SLA-1*0801 [20,32]. All candidate epitopes identified herein, have MHC IC_50_ values ≤500 nmol/dm^3^ and are classified as good binders [22]. Another significant property that must be considered in any drug and vaccine development procedures is safety. Retrieved epitopes were filtered by conducting *in silico* toxin-peptide mapping to determine potential toxic epitopes; and Protein BLAST to identify epitopes with significant match to the sequences of *Sus scrofa* proteome in databases. Short peptides with e-values <1.0e-30 can be cross-reactive [23]. Candidate epitopes identified in this work can be classified as non-toxic. BLASTp assessments showed that the epitopes have e-values ranging from 0.19 to 75, making it less likely to cause cell damage and autoimmune reactions in swine. This study identified seven peptides from a total of 26 candidate cytotoxic T-cell epitopes that can promiscuously bind to at least two of the most widely distributed SLAs in swine. Promiscuous epitopes include IADAINQEF, FLNKSTQAY, QIYKTLLEY, and SLYPTQFDY in pp220; and GTDLYQSAM, FINSTDFLY, and STDFLYTAI in pp62. These cytotoxic T-cell peptides can potentially cover wider range of swine populations around the world, thereby increasing peptide vaccine immunogenicity. Moreover, resulting values of binding affinity (K_d_) which are less than 1.0 and the negative binding energy (DG_bind_) of the candidate epitopes P1, P2, P3, and P4 with SLA-1*0401 (Table-3), indicate favorable complex formation and validate the cytotoxic T-cell epitope mapping conducted in this study. To further demonstrate the immunogenicity of the candidate epitopes, the stability of peptide-SLA complex was evaluated by plotting the RMSD per residue which often represents the mobility of a residue in molecular dynamics simulation. More stable interactions have weaker mobility; thus, lower RMSD value. The RMSD values of all the residues in each peptide-SLA complex formed range from 0.0 to 0.9 Ǻ, indicating stable interactions [33]. This is despite the use of the weakest (with the highest MHC IC_50_) cytotoxic T-cell epitopes (P1, P2, P3, and P4) from pp220, pp62, p72, and p30 in the docking procedures and molecular dynamics evaluations with SLA-1*0401. In addition, the value of K_d_, DG_bind_, and RMSD plot of influenza A H1N1 virus cytotoxic T-cell epitope bound to the crystal structure of SLA-1*0401 was also evaluated as a control, to further highlight the positive binding of identified candidate cytotoxic T-cell epitopes to SLA. Table-3 and Figure-2 show that the binding affinity, binding energy, and RMSD plot of influenza A H1N1 epitope are comparable to the values estimated for candidate epitopes predicted in this work.

### Limitations of the study

This study has provided preliminary data for the potential immunotherapeutic targets against ASFV. A computational approach was conducted in the analysis of highly conserved sequences and epitopes mapped from the structural proteins of ASFV. In addition, only cytotoxic T-cell epitopes of ASFV pp220, pp62, p72, p30, and CD2v were covered in this study. B-cell and helper T-cell epitopes can also be analyzed from the identified highly conserved sequences in succeeding studies. As with the step-by-step processes in any vaccine development, it is anticipated that *in vitro* and *in vivo* assays will be conducted in the future before application of the candidate epitopes inferred in this study.

## Conclusion

This is the first work to identify highly conserved cytotoxic T-cell epitopes in the structural proteins pp220, pp62, p72, p30, and CD2v which have important roles in the virulence of ASFV. *In silico* analysis showed that the candidate epitopes can be safely used in vaccine formulations as they are classified non-toxic and are less likely to cross-react with the domestic swine proteome. The peptides can potentially cover wider domestic swine populations and can bind to their corresponding SLA alleles with stability which indicates that the epitopes are potentially immunogenic. Finally, highly conserved cytotoxic T-cell epitopes identified in this study may avoid immune evasion to offer longer and more effective protection against ASFV infection. The use of these candidate epitopes as part of future peptide or recombinant vaccines is anticipated to be validated both *in vitro* and *in vivo* in subsequent studies.

## Authors’ Contributions

LRMH: Conceived the study. LRMH and EPB: Drafted the manuscript and analyzed the data. Both authors revised the manuscript and approved the final manuscript.
